# Supplementary material for: From Static to Dynamic Structures: Improving Binding Affinity Prediction with Graph‐Based Deep Learning
Source: Adv Sci (Weinh). 2024 Aug 29;11(40):2405404. doi: 10.1002/advs.202405404 (PMC11516055; doi:10.1002/advs.202405404)
Supplement: Supplementary file 1 — Supporting Information [file ADVS-11-2405404-s001.pdf]

ADVANCED SCIENCE
Open Access

Supporting Information

for *Adv. Sci.*, DOI 10.1002/advs.202405404

From Static to Dynamic Structures: Improving Binding Affinity Prediction with Graph-Based Deep Learning

Yaosen Min, Ye Wei, Peizhuo Wang, Xiaoting Wang, Han Li, Nian Wu, Stefan Bauer, Shuxin Zheng, Yu Shi, Yingheng Wang, Ji Wu*, Dan Zhao* and Jianyang Zeng**

Supplementary Information:

From Static to Dynamic Structures: Improving Binding Affinity Prediction with Graph-Based Deep Learning

Yaosen Min [1,†], Ye Wei [1,†,*], Peizhuo Wang [1,2,†], Xiaoting Wang [3], Han Li [1], Nian Wu [1], Stefan Bauer [4], Shuxin Zheng [5], Yu Shi [5], Yingheng Wang [6], Ji Wu [6,*], Dan Zhao [1,*], and Jianyang Zeng [7−9,*]

1 Institute for Interdisciplinary Information Sciences, Tsinghua University, 100084, Beijing, China.
2 School of Life Science and Technology, Xidian University, 710071, Xi'an, Shaanxi, China.
3 School of Medicine, Tsinghua University, 100084, Beijing, China.
4 Department of Intelligent Systems, KTH, 10044, Stockholm, Sweden.
5 Microsoft Research Asia, 100080, Beijing, China.
6 Department of Electrical Engineering, Tsinghua University, 100084, Beijing, China.
7 School of Engineering, Westlake University, 310030, Hangzhou, China.
8 Research Center for Industries of the Future, Westlake University, 310030, Hangzhou, China.
9 Westlake Laboratory of Life Sciences and Biomedicine, Westlake University, 310024, Hangzhou, China.
† These authors contributed equally to this work.
∗ All correspondence should be addressed to: weiye@mail.tsinghua.edu.cn, wuji_ee@mail.tsinghua.edu.cn, zhaodan2018@tsinghua.edu.cn, and zengjy@westlake.edu.cn.

This document contains:

- Supplementary Notes
- Supplementary Tables S1-S4
- Supplementary Figures S1-S4

A. Supplementary Notes

A.1. Preparation of the molecular dynamics dataset

In this study, we used the CHARMM-GUI service (https://charmm-gui.org/) to prepare the input files for our MD simulations [1, 2]. Here, we provide supplementary details about the MD system preparation protocol, describing the steps and tools employed.

Initially, we input the PDB ID obtained from the PDBBind dataset [3] into the solution builder tool in the input generator page of CHARMM-GUI. We selected the "Check/Correct PDB Format" option to validate the input file. Next, we selected only the ligand and the interacting chain in the model/chain selection step, excluding any solvent molecules. The interacting chain was determined based on annotations from the RCSB PDB [4], using ligand interaction visualization for assistance. In the PDB manipulation step, we parameterized the ligand with the ParamChem service [5], utilizing the SDF file from the RCSB PDB. We fixed all missing residues using GalaxyFill [6] and left other options unselected. We employed a rectangular water box and added the KCl ions to neutralize the system, setting the concentration to 0.15M using the Monte-Carlo placing method [1, 2]. Periodic boundary conditions were used by default. We chose the CHARMM36 force field and selected NAMD as the MD engine.

The simulation times for equilibration (NVT ensemble) and production (NPT ensemble) were set to 0.25 ns and 10 ns, respectively, with a temperature of 303.15 K. Finally, we downloaded the input files and used the provided script to run the simulations. The simulations were run on the NVIDIA V100 GPU, and all simulation jobs were completed within 3 months.

A.2. Details on the model architecture

The backbone of Dynaformer is based on the encoder of a vanilla transformer [7]. More specifically, inspired by Graphormer [8], a variant of transformer designed for encoding molecular graphs, we modified the multi-head self-attention module to encode the structural interaction information of protein-ligand complexes. Given an input graph $\mathcal{G} = (\mathcal{V}, \mathcal{E})$, we denote the node set as $\mathcal{V} = \{\mathbf{v}_i \mid 0 \leq i \leq n\} \in \mathbb{R}^{(n+1)\times 9}$, representing the input features of n atoms and a virtual node $\mathbf{v}_0$. The edge set is denoted as $\mathcal{E} = \{\mathbf{e}_{i,j} \mid i, j \in \mathcal{V}\} \in \mathbb{R}^{m\times 3}$, representing the input features of m covalent or non-covalent interactions between atoms (Supplementary Table S1). Similarly, we denote the hidden representations with dimension d_h of all $(n+1)$ nodes as $\mathbf{H} = \{\mathbf{h}_i \mid 0 \leq i \leq n\} \in \mathbb{R}^{(n+1)\times d_h}$.

Briefly, our Dynaformer model is composed of multiple stacked Dynaformer layers, each consisting of a multi-head self-attention (MHA) module and a feed-forward network (FFN) module. In the MHA module, the self-attention mechanism computes a weighted sum of input node representations, with weights determined by the attention scores between node representations. Here, each head of the attention mechanism can be defined as follows:

$$\text{Attention}(\mathbf{Q}, \mathbf{K}, \mathbf{V}) = \text{softmax}(\mathbf{A}) \cdot \mathbf{V}, \tag{1}$$

$$\mathbf{Q} = \mathbf{H}\mathbf{W_Q}, \mathbf{K} = \mathbf{H}\mathbf{W_K}, \mathbf{V} = \mathbf{H}\mathbf{W_V}, \tag{2}$$

$$A_{ij} = \frac{(\mathbf{h}_i\mathbf{W_Q})(\mathbf{h}_i\mathbf{W_K})^T}{\sqrt{d_K}} + d(i,j) + a(i,j) + \frac{1}{P}\sum_{p=1}^{P} \mathbf{e}_p \mathbf{w}_p^T, \tag{3}$$

where $\mathbf{W}_Q \in \mathbb{R}^{d_h, d_K}$, $\mathbf{W}_K \in \mathbb{R}^{d_h, d_K}$, and $\mathbf{W}_V \in \mathbb{R}^{d_h, d_K}$ stand for the learnable parameters, d_K stands for the shared last dimension of query matrix $\mathbf{Q}$, key matrix $\mathbf{K}$ and value matrix $\mathbf{V}$, and $\mathbf{A} \in \mathbb{R}^{(n+1)\times(n+1)}$ stands for the attention score matrix, with $A_{ij} \in \mathbb{R}$ denoting the attention score between nodes i and j. The attention score A_{ij} is determined by four terms. The first term $\frac{(\mathbf{h}_i\mathbf{W_Q})(\mathbf{h}_i\mathbf{W_K})^T}{\sqrt{d_K}}$ is the scaled dot-product attention, which is the same as in the vanilla transformer [7]. The second term $d(i,j)$ and the third term $a(i,j)$ encode the distance and angle features between atoms i and j using the Gaussian basis function (GBF), respectively. The last term $\frac{1}{P}\sum_{p=1}^{P} \mathbf{e}_p \mathbf{w}_p^T$ encodes the inter-atomic interactions on the shortest path up to length P between atoms i and j, where $\mathbf{w}_p$ stands for the weight that projects the edge feature vector $\mathbf{e}_p$ to a scalar. In this work, the last three terms in the attention score function are called the structural encodings, as they mainly inform the model about the structural information of a protein-ligand complex. Furthermore, the distance encoding function $d(i,j)$ and the angle encoding function $a(i,j)$ can be defined as follows:

$$\begin{aligned} \text{GBF}(x_k) &= \exp\left[-\frac{(x_k - \mu_k)^2}{2\sigma_k^2}\right], k = 1, \ldots, K, \\ d(i,j) &= \text{GBF}\left([\mathbf{v}_i | r_{ij} | \mathbf{v}_j]\,\mathbf{W}_d\right), \\ a(i,j) &= \text{GBF}\left(\left[\mathbf{v}_i | \sum_k \angle ijk | \mathbf{v}_j\right]\mathbf{W}_a\right), \end{aligned} \tag{4}$$

where μ_k and σ_k stand for the learnable parameters, K stands for the number of encoding heads in the GBF function, x_k stands for a distance or angle feature to be encoded, $\mathbf{v}_i$ stands for the atomic features of atom i, $|$ represents the concatenation operation between vectors, r_{ij} stands for the Euclidean distance scalar in angstrom between atoms i and j, $\angle ijk$ signifies the angle scalar in degree between bonds formed among atoms i, j, and k, and $\mathbf{W}_d$ and $\mathbf{W}_a$

correspond to the weight matrices for distance and angle features, respectively. Then, our MHA module is defined as a concatenation of h heads of the single-head self-attention:

$$\mathrm{MHA}\left(\mathbf{H}\right) = \mathrm{Concat}\left(\mathrm{head}_1, \ldots, \mathrm{head}_h\right)\mathbf{W}_{\mathbf{C}}, \tag{5}$$

$$\mathbf{Q}_i = \mathbf{H}\mathbf{W}_{\mathbf{Q},i}, \mathbf{K}_i = \mathbf{H}\mathbf{W}_{\mathbf{K},i}, \mathbf{V}_i = \mathbf{H}\mathbf{W}_{\mathbf{V},i}, \tag{6}$$

$$\mathrm{head}_i = \mathrm{Attention}\left(\mathbf{Q}_i, \mathbf{K}_i, \mathbf{V}_i\right), \tag{7}$$

where $\mathbf{W}_{\mathbf{Q},i}$, $\mathbf{W}_{\mathbf{K},i}$, and $\mathbf{W}_{\mathbf{V},i}$ stand for the learnable parameters of the i-th self-attention head to produce $\mathbf{Q}_i$, $\mathbf{K}_i$, and $\mathbf{V}_i$ matrices, respectively, and $\mathbf{W}_C$ stands for another learnable parameter for the linear transformation of multiple heads to the original hidden space of dimension d_h.

Our FFN module consists of two fully connected layers with a ReLU activation in between. More specifically, our FFN module can be defined as follows:

$$\mathrm{FFN}(\mathbf{H}) = \mathrm{ReLU}\left(\mathbf{H}\mathbf{W}_1 + \mathbf{b}_1\right)\mathbf{W}_2 + \mathbf{b}_2, \tag{8}$$

where $\mathbf{W}_1 \in \mathbb{R}^{d_h \times d_h}$, $\mathbf{W}_2 \in \mathbb{R}^{d_h \times d_h}$ stand for the learnable weight matrices, and $\mathbf{b}_1 \in \mathbb{R}^{d_h}$, $\mathbf{b}_2 \in \mathbb{R}^{d_h}$ stand for the bias vectors. In addition, layer normalization (LN) is applied to normalize the hidden representations [9]. The LN can be defined as:

$$\mathrm{LN}(\mathbf{H}) = \frac{\mathbf{H} - \mu(\mathbf{H})}{\sigma(\mathbf{H})} \odot \gamma + \beta, \tag{9}$$

where $\mu(\mathbf{H})$ and $\sigma(\mathbf{H})$ stand for the mean and standard deviation values of $\mathbf{H}$, respectively, and γ and β stand for the learnable parameters.

In total, each layer of our Dynaformer model consists of an MHA module and an FFN module to convert the hidden representations $\mathbf{H}^{(l)}$ of the l-th layer to the hidden representations $\mathbf{H}^{(l+1)}$ of the $(l+1)$-th layer. Note that $\mathbf{H}^{(0)}$ is the embedding of the input node feature $\mathcal{V}$. The operations in a Dynaformer layer can be defined as:

$$\begin{aligned}\hat{\mathbf{H}}^{(l)} &= \mathrm{MHA}\left(\mathbf{H}^{(l-1)}\right), \\ \hat{\mathbf{H}}'^{(l)} &= \mathrm{LN}\left(\hat{\mathbf{H}}^{(l)} + \mathbf{H}^{(l-1)}\right), \\ \mathbf{H}^{(l+1)} &= \mathrm{LN}\left(\mathrm{FFN}\left(\hat{\mathbf{H}}'^{(l)}\right) + \hat{\mathbf{H}}'^{(l)}\right),\end{aligned} \tag{10}$$

where $\hat{\mathbf{H}}^{(l)}$ and $\hat{\mathbf{H}}'^{(l)}$ stand for the intermediate hidden representations of the l-th layer. After the final layer, the hidden representation $\mathbf{H}^{(L)}$ of the last layer is used to yield the output

of the model. More specifically, after L layers, the representation of the virtual node $\mathbf{h}_0^{\prime(L)}$ is extracted as the global pooled representation of the graph, and it is then concatenated with three linear transformed fingerprint vectors to predict the binding affinity of the input graph. That is, the prediction can be defined as follows:

$$\begin{aligned} \mathbf{h}_0^{\prime(L)} &= \text{Concat}\left(\mathbf{h}_0^{(L)}, \text{Concat}\left(\mathbf{fp}_1, \mathbf{fp}_2, \mathbf{fp}_3\right)\mathbf{W}_{fp}\right), \\ y &= \mathbf{h}_0^{\prime(L)}\mathbf{W}_y + \mathbf{b}_y, \end{aligned} \tag{11}$$

where $\mathbf{W}_{fp} \in \mathbb{R}^{d_{fp}\times d_h}$, $\mathbf{W}_y \in \mathbb{R}^{2d_h\times 1}$, and $\mathbf{b}_y \in \mathbb{R}$ stand for the learnable parameters, d_{fp} stands for the dimension of all fingerprint vectors, $\mathbf{h}_0^{\prime(L)}$ stands for the intermediate representation for prediction, and $y \in \mathbb{R}$ stands for the predicted binding affinity of the input graph.

A.3. Description and evaluation of the baseline methods

According to their characteristics, we divided the existing methods into four types: docking-based, feature-based, voxel-based, and graph-based methods. A comprehensive performance evaluation of Dynaformer and the baseline methods is presented in Supplementary Table S3. The performances of the methods marked with asterisks were directly obtained from SIGN's paper [10]. Brief descriptions of the baseline methods are provided below.

Docking-based methods. This type of method uses an energy function to guide the searching algorithm to find the optimal position of a ligand binding to a target protein, and the energy of the optimal position is used as the predicted binding affinity. Several well-known energy functions and docking software are tested in this paper. More specifically, GoldScore, ChemPLP, and ASP are scoring functions implemented in the GOLD docking software [11–13], GlideScore-XP and GlideScore-SP are two variants of GlideScore packed with GLIDE (version 5.0) [14] and Autodock Vina is the docking energy function implemented within the Autodock Vina software [15].

Feature-based methods. This type of method uses a certain set of elaborate features and trains different machine learning models, such as random forests or gradient boosting decision trees, to predict binding affinities. Here, the feature-based scoring functions heavily rely on the designer's expertise in protein-ligand binding, and thus performance often varies widely between different methods. DrugScore2018 is a scoring function that is derived based on statistical potentials and uses atom types and distances to calculate the binding free energy with a knowledge-based potential function [16]. X-Score and its variants, X-ScoreHM, X-ScoreHP, and X-ScoreHS, are empirical scoring functions that are proposed to account for

various types of atomic interactions [17]. ΔSAS uses the difference in the solvent accessible surface (SAS) of the ligand before and after binding to the protein as the sole feature for prediction [18]. The RF-Score utilizes atom pair counts within a defined distance cutoff, as input features. It then employs a random forest algorithm to predict the binding affinity between proteins and ligands [19]. ΔVinaRF20 is a scoring function that uses random forests to predict the correction term of the binding energies calculated from Autodock Vina. It contains 20 descriptors derived from experimental structures [20]. ECIF uses pairs of different protein-ligand atom-types and a set of ligand molecular descriptors to describe protein-ligand complexes and build a gradient boosting decision tree to predict the corresponding binding affinities [21]. Multi-shell ECIF (MSECIF) enhances ECIF by introducing distance-based virtual shells. These shells segment inter-atomic distances into zones, with interaction counts within each zone serving as distinct features [22].

Voxel-based methods. This type of method treats protein-ligand complexes as 3D grids in space and computes the features of molecular segments within each 3D voxel as input data for the 3D CNN models. Pafnucy is a deep neural network model that utilizes a 3D convolution neural network to encode the feature map of a 3D grid-represented complex for predicting binding affinities [23]. Kdeep applies a similar strategy and utilizes a 3D convolution neural network for prediction. The difference between Pafnucy and Kdeep is their initial feature design and detailed model architectures [24]. OnionNet combines the feature-based and voxel-based methods by first calculating the onion-like feature layers of the binding environment, and then applying a convolution neural network to encode the features for prediction [25].

Graph-based methods. This type of method considers molecules as graphs and uses graph neural networks (GNN) to achieve rotation invariance and make predictions. In particular, SGCN is built on a graph convolution network (GCN) that uses spatial features to learn graph representations [26]. GraphDTA uses graph neural network models to learn the feature representations of graph-structured data and utilizes a convolution neural network to learn the feature representations of protein sequences [27]. Then these two types of features are fused for binding prediction. Directed message passing neural network (DMPNN) applies an edge-based message passing neural network to perform aggregation on edges instead of atoms to construct molecular embeddings [28]. GNN-DTI introduces a distance-aware graph neural network model to predict binding affinities from various types of intermolecular interactions based on the interaction graph [29]. Molecule attention transformer (MAT) uses the attention mechanism in a transformer based on the inter-atomic distances and the molecular graph structure [30]. DimeNet uses directional message passing and simultane-

ously considers atoms, interatomic distances, and three angles between chemical bonds in the message-passing phase [31]. Communicative message passing neural network (CMPNN) further extends DMPNN and aims to enhance molecular embedding by using a communicative kernel to strengthen the message interactions between nodes and edges [32]. SIGN proposes a structure-aware interactive graph neural network to predict binding affinities. It iteratively performs the node-edge aggregation process to update the feature representations of nodes and edges while preserving the distance and angle information among atoms [10].

B. Supplementary Tables

Supplementary Table S1. Features of input graph. The protein-ligand complex structures are processed into graph representations containing the following atom (node) and interatomic (edge) features.

Type	Feature name	Valid value
Node feature	Atomic number	[1, 2, ..., 119, misc]
	Chirality	[R, S, Other, Unspecified]
	Degree	[0, 1, ..., 10, misc]
	Formal charge	[-5, -4, ..., 5, misc]
	Num. hydrogen atoms	[0, ..., 8, misc]
	Num. radical electrons	[0, 1, 2, 3, 4, misc]
	Hybridization	[sp, sp^2, sp^3, sp^3d, sp^3d^2, misc]
	Aromaticity	[True, False]
	Is in ring	[True, False]
Edge feature	Bond type	[Single, Double, Triple, Aromatic, misc]
	Bond stereo	[Z, E, cis, trans, None, misc]
	Is conjugated	[True, False]

Supplementary Table S2. Model configuration and pretraining hyperparameters of Dynaformer.

Name	Value
#Layers	4
Hidden dimension	512
FFN Inner-layer Dimension	512
#Attention Heads	32
Hidden Dimension of Each Head	16
FFN Dropout	0.1
Attention Dropout	0.1
Embedding Dropout	0.1
Max Epochs	100
Peak Learning Rate	1e-4
Batch size	160
Warm-up Steps	20,000
Learning Rate Decay	Linear
Adam ϵ	1e-8
Adam (β_1, β_2)	(0.9, 0.999)
Weight Decay	1e-5
FLAG #Steps	3
FLAG Stepsize	0.001
FLAG Noise Magnitude	0.01

Supplementary Table S3. Detailed performance evaluation of Dynaformer and baseline methods. ↑ represents higher the better and ↓ represents lower the better.

Method		Scoring Power			Ranking Power		
		PR ↑	SD ↓	RMSE ↓	SR ↑	KT ↑	PI ↑
**Docking-based Methods**	GoldScore	0.416	1.991	1.986	0.284	0.242	0.283
	GlideScore-XP	0.467	1.947	1.944	0.257	0.227	0.255
	GlideScore-SP	0.513	1.890	1.887	0.419	0.374	0.425
	AutodockVina	0.604	1.733	1.729	0.528	0.453	0.557
	ChemPLP	0.614	1.723	1.720	0.633	0.537	0.657
	ASP	0.617	1.714	1.711	0.553	0.474	0.582
**Feature-based Methods**	DrugScore2018	0.602	1.736	4.240	0.607	0.530	0.637
	X-ScoreHM	0.609	1.725	1.722	0.603	0.522	0.641
	X-ScoreHP	0.621	1.704	1.701	0.573	0.508	0.607
	ΔSAS	0.625	1.697	1.698	0.588	0.498	0.612
	X-ScoreHS	0.629	1.690	1.687	0.547	0.469	0.577
	X-Score	0.631	1.688	1.685	0.604	0.529	0.638
	RF-Score	0.801	1.301	1.299	0.602	0.527	0.631
	ΔVinaRF20	0.816	1.256	1.253	0.750	0.686	0.761
	ECIF	0.845	1.162	1.160	0.726	0.625	0.752
	MSECIF	0.859	1.114	1.197	0.714	0.625	0.746
**Voxel-based Methods**	Pafnucy	0.769	1.390	1.388	0.600	0.511	0.627
	Kdeep	0.803	1.297	1.295	0.670	0.575	0.693
	OnionNet	0.813	1.268	1.266	0.657	0.559	0.693
**Graph-based Methods**	SGCN*	0.686	1.582	1.583	/	/	/
	GraphDTA*	0.699	1.558	1.562	/	/	/
	DMPNN*	0.729	1.489	1.493	/	/	/
	GNN-DTI*	0.736	1.471	1.492	/	/	/
	MAT*	0.747	1.445	1.457	/	/	/
	DimeNet*	0.752	1.434	1.453	/	/	/
	CMPNN*	0.765	1.399	1.408	/	/	/
	SIGN	0.788	1.341	1.338	0.598	0.518	0.622
	Dynaformer	0.858	1.116	1.114	0.767	0.684	0.788

Supplementary Table S4. Ablation study of Dynaformer. F stands for the incorporation of fingerprints, 3D stands for the structural feature encoding module, and M stands for the pretraining on the MD trajectory dataset.

Components			Scoring Power			Ranking Power		
F	3D	M	PR ↑	SD ↓	RMSE ↓	SR ↑	KT ↑	PI ↑
✓	✓	✓	0.858	1.114	1.116	0.767	0.684	0.788
✗	✓	✓	0.849	1.152	1.149	0.746	0.649	0.769
✓	✗	✓	0.836	1.192	1.193	0.712	0.607	0.735
✓	✓	✗	0.827	1.266	1.221	0.665	0.575	0.682
✗	✗	✓	0.841	1.179	1.178	0.723	0.625	0.745
✓	✗	✗	0.786	1.463	1.344	0.646	0.551	0.680
✗	✓	✗	0.806	1.303	1.286	0.621	0.533	0.645
✗	✗	✗	0.731	1.480	1.483	0.553	0.460	0.579

C. Supplementary Figures

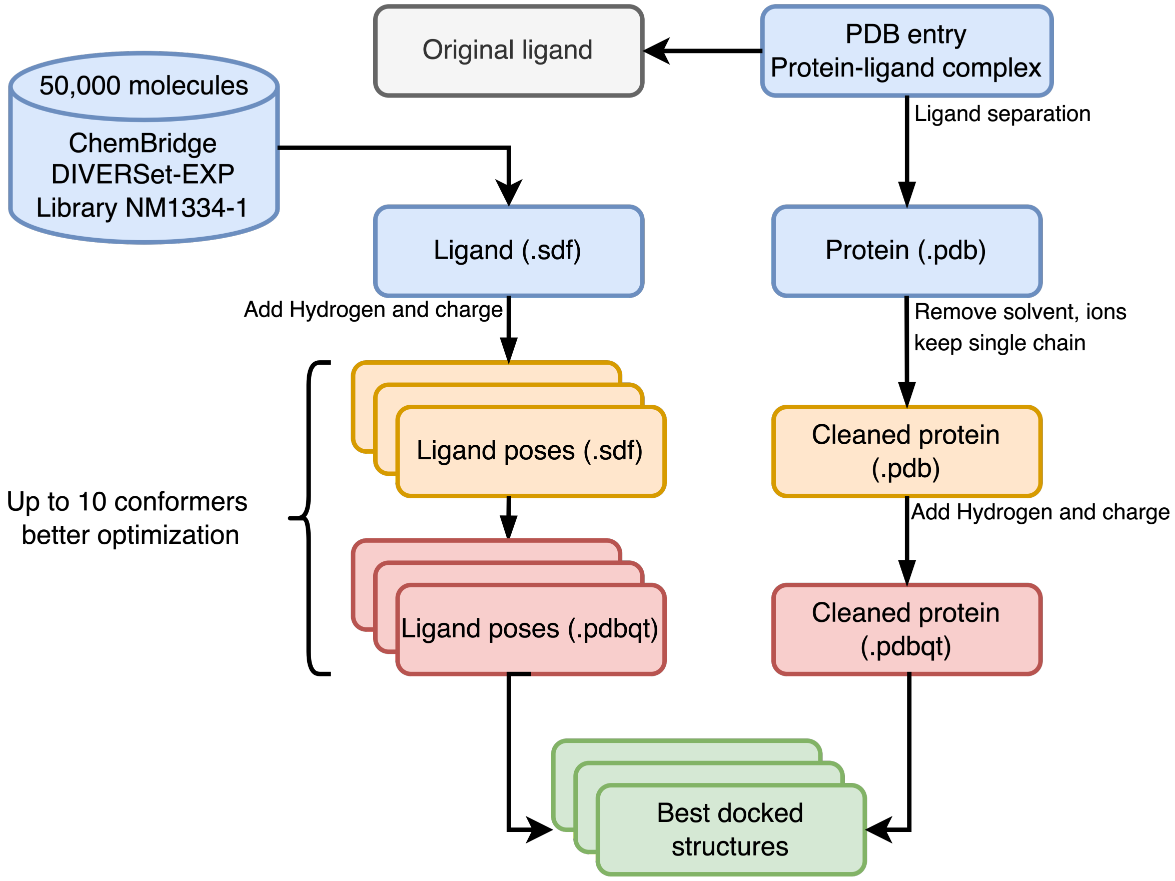

Supplementary Figure S1. The pipeline of ligand preparation, receptor preparation, and docking with Autodock Vina.

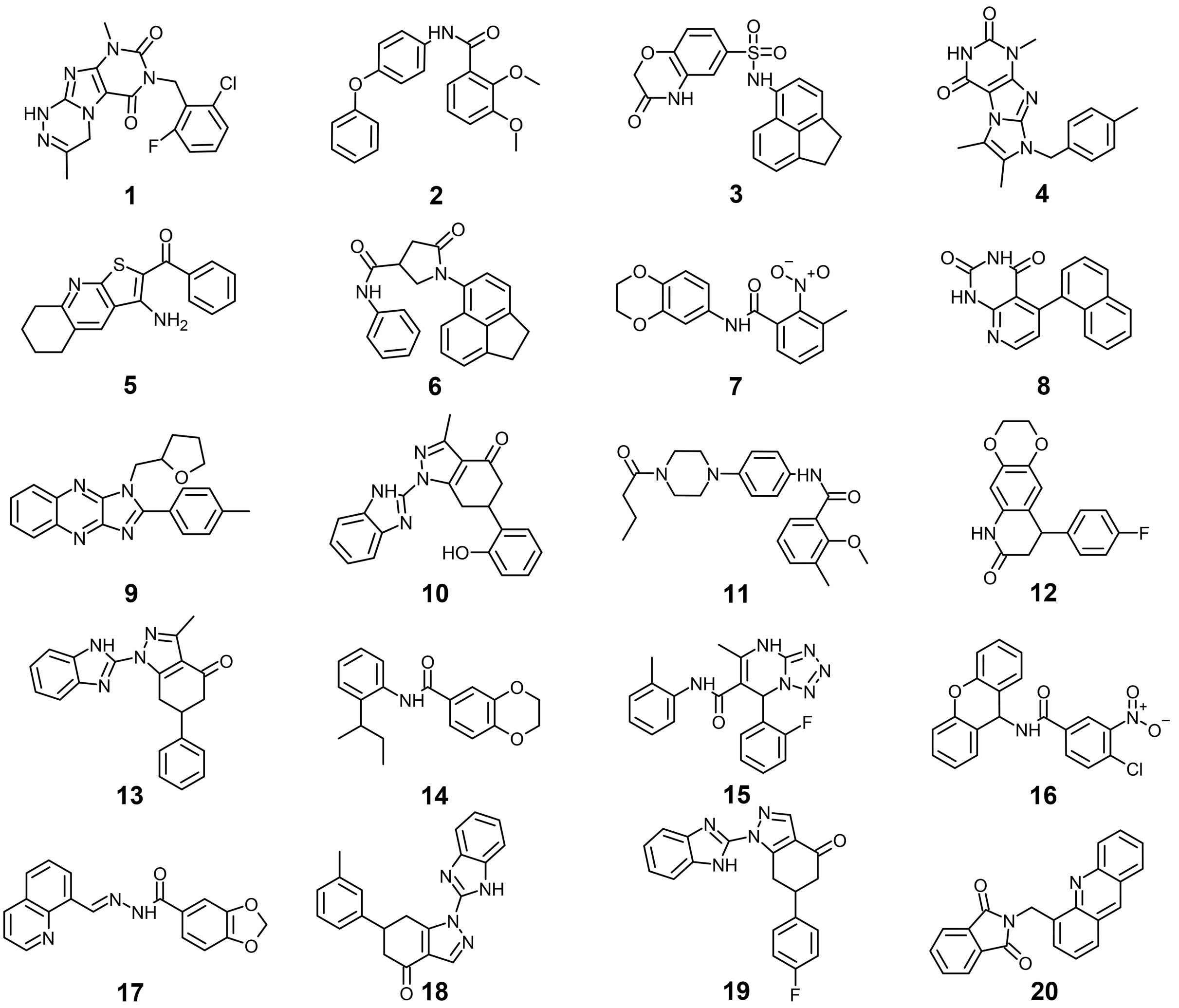

Supplementary Figure S2. Compounds selected from virtual screening for experiment validation.

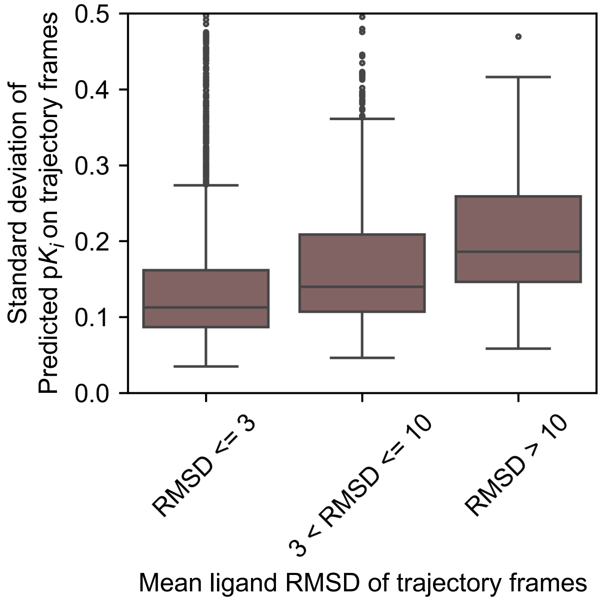

Supplementary Figure S3. The box plot of standard deviation of predicted pK_i values and the mean ligand RMSD.

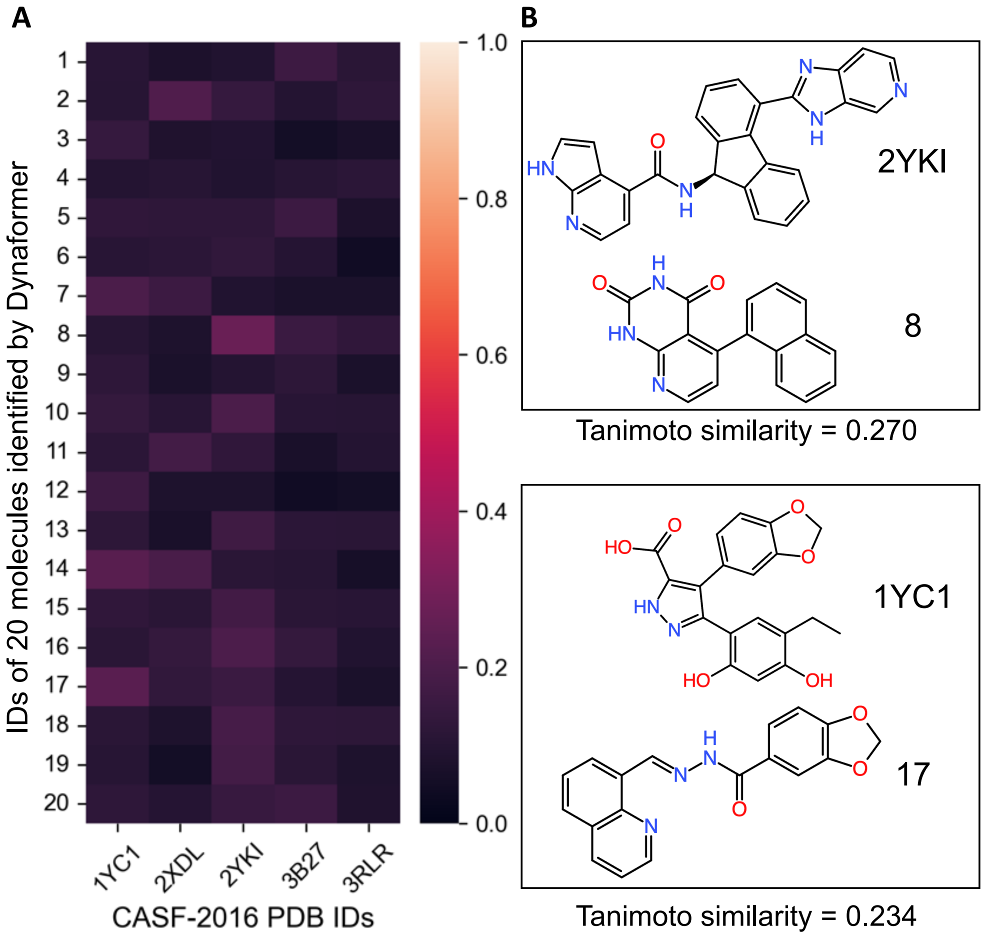

Supplementary Figure S4. Molecular similarity between HSP90 ligands in CASF-2016 and hit compounds from our virtual screening. **A**) Tanimoto similarity matrix between ECFP-4 fingerprints of the compared molecules. The 2XDL (pK_i = 3.10) that possesses the lowest binding affinity was used as the reference structure for docking. **B**) Structures of the two most similar pairs of molecules shown in the similarity matrix.

References

[1] Jo, S., Kim, T., Iyer, V. G. & Im, W. Charmm-gui: a web-based graphical user interface for charmm. *Journal of computational chemistry* **29** (11), 1859–1865 (2008) .

[2] Lee, J. *et al.* Charmm-gui input generator for namd, gromacs, amber, openmm, and charmm/openmm simulations using the charmm36 additive force field. *Journal of chemical theory and computation* **12** (1), 405–413 (2016) .

[3] Wang, R., Fang, X., Lu, Y. & Wang, S. The pdbbind database: Collection of binding affinities for protein- ligand complexes with known three-dimensional structures. *Journal of medicinal chemistry* **47** (12), 2977–2980 (2004) .

[4] Berman, H. M. *et al.* The protein data bank. *Nucleic acids research* **28** (1), 235–242 (2000) .

[5] Ghosh, J. *et al.* Molecular parameter optimization gateway (paramchem): Workflow management through teragrid asta. *TeraGrid Conference: Extreme Digital Discovery* (2011) .

[6] Ko, J. *et al.* The falc-loop web server for protein loop modeling. *Nucleic acids research* **39** (suppl_2), W210–W214 (2011) .

[7] Vaswani, A. *et al.* Attention is all you need. *Advances in neural information processing systems* **30** (2017) .

[8] Ying, C. *et al.* Do transformers really perform badly for graph representation? *Advances in Neural Information Processing Systems* **34**, 28877–28888 (2021) .

[9] Ba, J. L., Kiros, J. R. & Hinton, G. E. Layer normalization. *arXiv preprint arXiv:1607.06450* (2016) .

[10] Li, S. *et al.* Structure-aware interactive graph neural networks for the prediction of protein-ligand binding affinity. *ACM SIGKDD Conference on Knowledge Discovery & Data Mining* 975–985 (2021) .

[11] Verdonk, M. L., Cole, J. C., Hartshorn, M. J., Murray, C. W. & Taylor, R. D. Improved protein–ligand docking using gold. *Proteins: Structure, Function, and Bioinformatics* **52** (4), 609–623 (2003) .

[12] Korb, O., Stutzle, T. & Exner, T. E. Empirical scoring functions for advanced protein-ligand docking with plants. *Journal of chemical information and modeling* **49** (1), 84–96 (2009) .

[13] Mooij, W. T. & Verdonk, M. L. General and targeted statistical potentials for protein–ligand interactions. *Proteins: Structure, Function, and Bioinformatics* **61** (2), 272–287 (2005) .

[14] Friesner, R. A. *et al.* Glide: a new approach for rapid, accurate docking and scoring. 1. method and assessment of docking accuracy. *Journal of medicinal chemistry* **47** (7), 1739–1749 (2004) .

[15] Trott, O. & Olson, A. J. Autodock vina: improving the speed and accuracy of docking with a new scoring function, efficient optimization, and multithreading. *Journal of computational chemistry* **31** (2), 455–461 (2010) .

[16] Dittrich, J., Schmidt, D., Pfleger, C. & Gohlke, H. Converging a knowledge-based scoring function: Drugscore2018. *Journal of chemical information and modeling* **59** (1), 509–521 (2018) .

[17] Wang, R., Lai, L. & Wang, S. Further development and validation of empirical scoring functions for structure-based binding affinity prediction. *Journal of computer-aided molecular design* **16** (1), 11–26 (2002) .

[18] Su, M. *et al.* Comparative assessment of scoring functions: the casf-2016 update. *Journal of chemical information and modeling* **59** (2), 895–913 (2018) .

[19] Ballester, P. J. & Mitchell, J. B. A machine learning approach to predicting protein–ligand binding affinity with applications to molecular docking. *Bioinformatics* **26** (9), 1169–1175 (2010) .

[20] Wang, C. & Zhang, Y. Improving scoring-docking-screening powers of protein–ligand scoring functions using random forest. *Journal of computational chemistry* **38** (3), 169–177 (2017) .

[21] Sánchez-Cruz, N., Medina-Franco, J. L., Mestres, J. & Barril, X. Extended connectivity interaction features: improving binding affinity prediction through chemical description. *Bioinformatics* **37** (10), 1376–1382 (2021) .

[22] Shiota, K. & Akutsu, T. Multi-shelled ecif: improved extended connectivity interaction features for accurate binding affinity prediction. *Bioinformatics Advances* **3** (1), vbad155 (2023) .

[23] Stepniewska-Dziubinska, M. M., Zielenkiewicz, P. & Siedlecki, P. Development and evaluation of a deep learning model for protein–ligand binding affinity prediction. *Bioinformatics* **34** (21), 3666–3674 (2018) .

[24] Jiménez, J., Skalic, M., Martinez-Rosell, G. & De Fabritiis, G. Kdeep: protein-ligand absolute binding affinity prediction via 3d-convolutional neural networks. *Journal of chemical information and modeling* **58** (2), 287–296 (2018) .

[25] Zheng, L., Fan, J. & Mu, Y. Onionnet: a multiple-layer intermolecular-contact-based convolutional neural network for protein–ligand binding affinity prediction. *ACS omega* **4** (14), 15956–15965 (2019) .

[26] Danel, T. *et al.* Spatial graph convolutional networks. *International Conference on Neural Information Processing* 668–675 (2020) .

[27] Nguyen, T. *et al.* Graphdta: Predicting drug–target binding affinity with graph neural networks. *Bioinformatics* **37** (8), 1140–1147 (2021) .

[28] Yang, K. *et al.* Analyzing learned molecular representations for property prediction. *Journal of chemical information and modeling* **59** (8), 3370–3388 (2019) .

[29] Lim, J. *et al.* Predicting drug–target interaction using a novel graph neural network with 3d structure-embedded graph representation. *Journal of chemical information and modeling* **59** (9), 3981–3988 (2019) .

[30] Maziarka, L. *et al.* Molecule attention transformer. *arXiv preprint arXiv:2002.08264* (2020) .

[31] Klicpera, J., Groß, J. & Günnemann, S. Directional message passing for molecular graphs. *arXiv preprint arXiv:2003.03123* (2020) .

[32] Song, Y. *et al.* Communicative representation learning on attributed molecular graphs. *International Joint Conference on Artificial Intelligence* (2021) .
